# Environmental conditions lead to shifts in individual communication, which can cause cascading effects on soundscape composition

**DOI:** 10.1002/ece3.9359

**Published:** 2022-10-01

**Authors:** Meelyn M. Pandit, Eli S. Bridge, Jeremy D. Ross

**Affiliations:** ^1^ Oklahoma Biological Survey University of Oklahoma Norman Oklahoma USA; ^2^ Department of Biology University of Oklahoma Norman Oklahoma USA

**Keywords:** agent‐based model, aridity, climate change, evaporative water loss, house finch, vocal communication

## Abstract

Climate change is increasing aridity in grassland and desert habitats across the southwestern United States, reducing available resources and drastically changing the breeding habitat of many bird species. Increases in aridity reduce sound propagation distances, potentially impacting habitat soundscapes, and could lead to a breakdown of the avian soundscapes in the form of loss of vocal culture, reduced mating opportunities, and local population extinctions. We developed an agent‐based model to examine how changes in aridity will affect both sound propagation and the ability of territorial birds to audibly contact their neighbors. We simulated vocal signal attenuation under a variety of environmental scenarios for the south, central semi‐arid prairies of the United States, ranging from contemporary weather conditions to predicted droughts under climate change. We also simulated how changes in physiological conditions, mainly evaporative water loss (EWL), would affect singing behavior. Under contemporary and climate change‐induced drought conditions, we found that significantly fewer individuals successfully contacted all adjacent neighbors than did individuals in either the contemporary or predicted climate change conditions. We also found that at higher sound frequencies and higher EWL, fewer individuals were able to successfully contact all their neighbors, particularly in drought and climate change drought conditions. These results indicate that climate change‐mediated aridification may alter the avian soundscape, such that vocal communication no longer effectively functions for mate attraction or territorial defense. As climate change progresses, increased aridity in current grasslands may favor shifts toward low‐frequency songs, colonial resource use, and altered songbird community compositions.

## Graphical abstract

Extreme contemporary and future arid conditions can lead to a degraded avian soundscape. Species with fixed singing behaviors are more likely to have a less functional vocal communication system than species with more flexible singing behaviors.

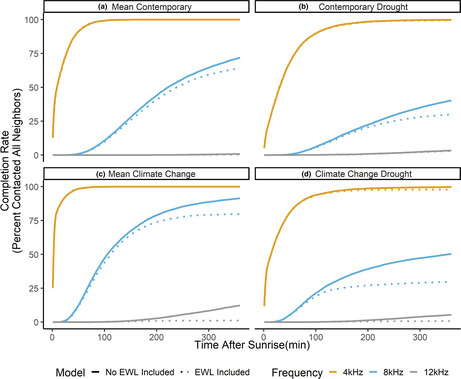

## 1 INTRODUCTION

Semi‐arid ecosystems are especially sensitive to climate change due to their relatively high temperatures, low precipitation, infertile soil, sparse vegetation cover, and low abundance of resources such as food and freshwater (Huang & Ullrich, 2017; Reynolds et al., 2007; Wei et al., 2019). As global warming intensifies, desertification and land degradation in dryland habitats are also predicted to increase (Huang et al., 2015). In the Mojave Desert, increases in temperature and aridity coupled with a decrease in water resources have led to the collapse of desert avian communities (Iknayan & Beissinger, 2018). While increases in temperature can lead to adaptations in heat dissipation (Song & Beissinger, 2019), with increasing aridity birds will also suffer from increased evaporative water loss. Understanding how increased desertification affects avian behavior may provide insight into community collapse and resiliency under new environmental conditions (Buchholz et al., 2019). In response to warmer temperatures, some birds advanced the onset of reproductive and singing behaviors to earlier dates in the breeding season (Dunn et al., 2010; Dunn & Møller, 2014; Rubolini et al., 2010), as well as shift the onset of singing to earlier in the day due to higher overnight temperatures (Bruni et al., 2014; Garson & Hunter Jr, 2008). Predicting how aridity changes birds' energy and water demand and how these changes may affect their communication behavior will be useful for understanding how other factors, such as reproduction, physiology, and even population recruitment, will be impacted by future weather conditions (du Plessis et al., 2012; Sharpe et al., 2019; Van de Ven et al., 2016).

Acoustic signals are used for intra‐ and interspecific communication among multiple animal species. Rapid changes in these signals can indicate that a population is adapting to environmental change associated with climate or other factors (Sueur & Farina, 2015). Since dry air is a poor sound conductor relative to moist air, we can expect increased aridity to degrade sound transmission fidelity. Henwood and Fabrick (1979) tested this theory by broadcasting acoustic signals of various frequencies (1.5, 3, and 6 kHz) in different environments at different times during the day. Broadcast coverage decreased dramatically across all signal frequencies when played in a desert environment in the afternoon compared to when played in a desert environment in the morning or compared to when played in a rainforest environment in the morning (Henwood & Fabrick, 1979). This effect is most pronounced for high‐frequency sounds, such as the songs of North American wood warblers, for which high‐frequency songs experienced high atmospheric attenuation (Snell‐Rood, 2012). For many bird species, the dawn chorus is a period of high vocalizing activity during the breeding season (Catchpole & Slater, 2008; Gil & Llusia, 2020; Staicer et al., 2020). This period is usually characterized by low temperatures and moderate‐to‐high humidity, which generally corresponds to the most optimal conditions for sound transmission (Henwood & Fabrick, 1979). Therefore, the dawn chorus may represent a behavioral adaptation that exploits the optimal sound transmission properties of early morning. Yet, climate models predict disproportionate nighttime temperature increases, suggesting that early morning conditions may become less optimal for vocal communication, potentially reducing the efficacy of the dawn chorus for adjacent neighbors to communicate (Mutiibwa et al., 2015). Birds may shift their pre‐dawn and dawn chorus start times earlier to sing during low‐light‐level periods, when foraging profitability is low due to low arthropod activity and constrained vision (Avery & Krebs, 1984; Kacelnik, 1979).

Increasing aridity will lead to reduced resources such as food and water (Reynolds et al., 2007, 2011), hence changing territory quality. To maintain suitable body conditions for survival and reproduction, individuals need to expand territory sizes to access enough resources (Khoury & Boulad, 2010). For example, in the Central Monte Desert in Argentina, rufous‐collared sparrows (*Zonotrichia capensis*) maintain larger breeding territories compared to their temperate or tropical counterparts during the breeding season due to the lower habitat quality of this arid region (Cecilia Sagario & Cueto, 2014).

Birds singing under increasingly arid conditions will not only simultaneously face poorer song transmission and territorial resource qualities, but the cost of singing itself will also increase, as the individual must shift allocations of time and energy resources away from singing (Reid, 1987; Zollinger & Brumm, 2015) and toward thermoregulatory and foraging behavior (du Plessis et al., 2012; Funghi et al., 2019; Gil & Gahr, 2002). Furthermore, aridity likely increases the water costs for singing birds due to accelerated evaporative water loss; singing exposes the high moisture gradient between the bird's respiratory tract and surrounding dry air, causing water loss to the environment (O'Connor et al., 2018; Ward et al., 2003; Ward & Slater, 2005). To avoid dehydration, birds will need to exhibit behavioral flexibility to sing at low dehydration conditions (Ducatez et al., 2020) or increase their rate of drinking (Czenze et al., 2020).

Agent‐based models (ABMs) are a powerful tool to analyze individual behaviors and their population‐level effects (Axelrod, 1997). ABMs are built around a set number of agents, defined behaviors, and rules; each agent's behavior is dependent on external stimuli fed into the model and the agent displays a behavior based on these stimuli and pre‐defined rules (Marceau, 2008; Reynolds, 1987). These models are useful in providing information on how behaviors can respond to future scenarios, such as increased temperature and aridity due to climate change, and on how the simulated system dynamics are affected. Studies used ABMs to predict migration start dates and routes for painted buntings (*Passerina ciris*; Bridge et al., 2015) and stopover duration and movement distances in North American dabbling ducks under changing weather conditions (Beatty et al., 2017). ABMs are a valuable tool in predicting how climate change will affect the behavior of individuals, and how those altered behaviors can affect the population (Patt & Siebenhüner, 2005). To our knowledge, ABMs have yet to map out how climate change will affect avian singing and territorial movement and how these changes will affect the avian soundscape.

**FIGURE 1:**
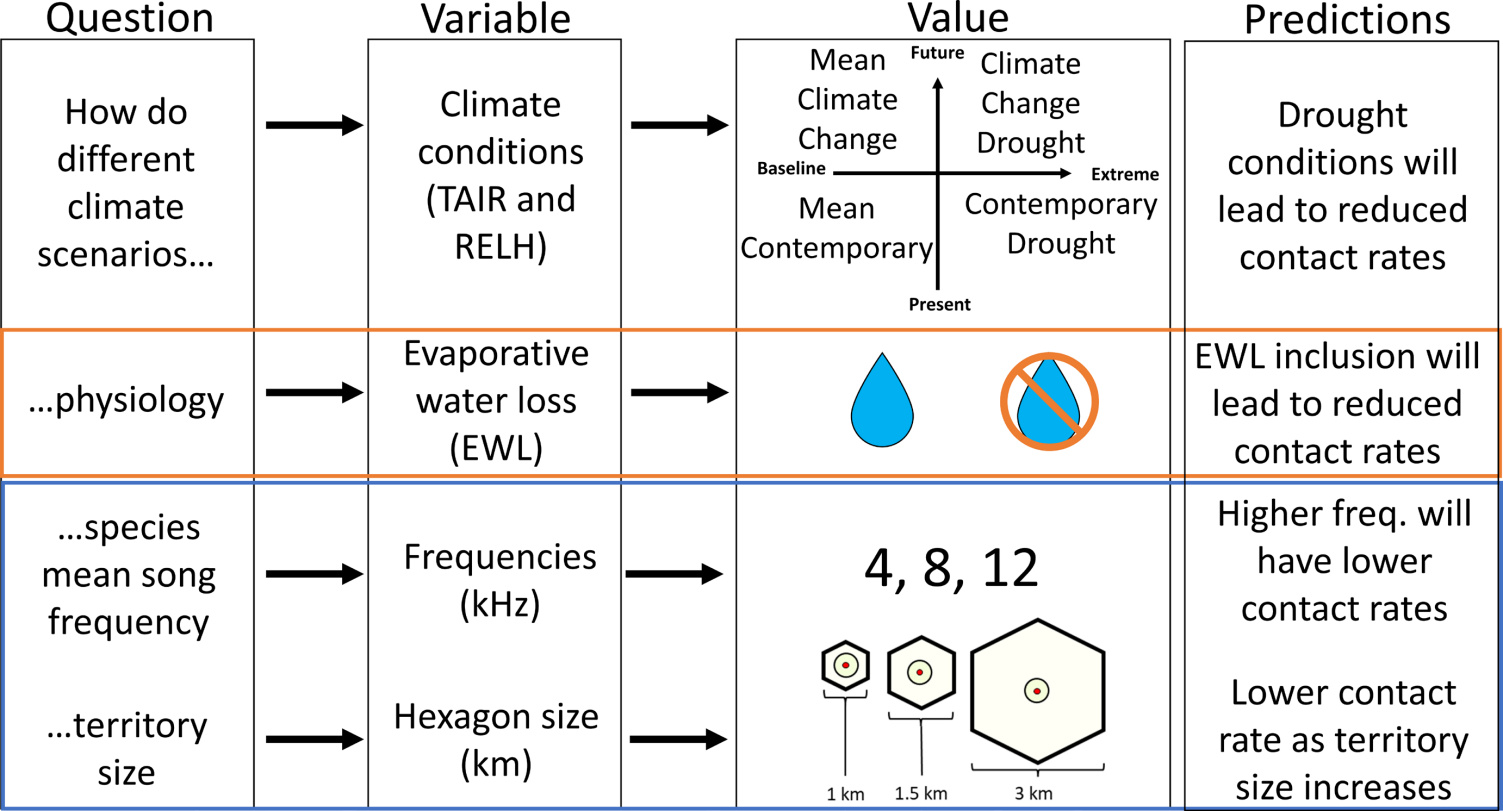
Pictogram of questions, hypotheses, and predictions for the ABM. The questions, variables, and values outlined in the orange box represent the FAB1 hypothesis, while the blue box represents the FAB2 hypothesis.

We examined how aridity would lead to a disrupted soundscape, and how this disrupted soundscape would affect avian singing, movement, and resting behavior. We developed two hypotheses to test how avian singing behavior changes under a disrupted soundscape: the facultative activity budget (FAB1) hypothesis, which states that singing activity is mainly dependent on individual conditions, and the fixed activity budget (FAB2) hypothesis which states that singing activity is fixed, and species‐specific traits are driving heterogeneity in vocal activity, regardless of individual condition. To evaluate these two hypotheses, we used an agent‐based model to simulate a population of individuals each with their own territories and their singing, movement, and resting behavior across contemporary and climate change‐induced weather conditions. We varied territory size and mean song frequency to determine which bird species would be most at‐risk of increased aridification (Figure 1).

We chose to simulate these disruptions under breeding conditions, both because alterations to breeding behavior would be more noticeable in an applied scenario and because the consequences of disruptions to breeding behavior have tangible consequences for recruitment and persistence. We predicted that individual contact rate would decrease with increasing frequency and territory size and that a population of singing individuals would not be able to maintain vocal contact with their immediate neighbors due to sound attenuation and reduced singing activity due to physiological constraints. We tested this hypothesis by using an ABM to model individual territorial vocalizations, within‐territory movement, and resting behavior across current and predicted climate conditions.

## 2 METHODS

### 2.1 Model design

We created an ABM in the program R v4.04 (R Core Team, 2021) that simulated the singing and movement behavior of individual “virtual birds” (from now on referred to as birds) within their respective territories. The model advanced at 1 min time steps, and at each time step every bird could perform one of three behaviors: sing, move within the territory, and rest. Decisions regarding which action was performed were determined by probabilities assigned to each action. At the first time step, the probability of each behavior was the same (0.33). The behavior probabilities only changed after a singing behavior was displayed or if the EWL equation was included in the model. When a singing behavior was initiated, a song radius was calculated based on the expected attenuation of the song. We set the threshold between audible and inaudible songs at 30 dB as the minimum amplitude that can be detected by the birds because it is the ambient sound amplitude (Yost, 2001). If a bird's song radius overlapped with the position of any of its neighbors, then a successful instance of communication was recorded for all individuals involved, and the neighbors within the song radius were induced to sing during that time step. If a bird's song radius did not overlap with its neighbor, the focal bird would either move or rest in the following time step, thereby changing the behavior probability to 0 for singing, 0.50 for moving, and 0.50 for resting. If a movement behavior was initiated, the bird would relocate to a random position within its territory, and if a resting behavior was initiated, the bird would take no action during the time step.

### 2.2 Experimental design

We ran the model using weather data from an Oklahoma Mesonet station in Western Oklahoma (ERIC) and we used these data to simulate future conditions caused by climate change (Brock et al., 1995; McPherson et al., 2007). We chose the ERIC Mesonet station because it has recorded some of the driest temperatures in the state of Oklahoma. We used weather data from May and June, a period that roughly corresponds to peak singing activity in birds. Each modeled day used weather data from sunrise until 6 h after sunrise which corresponded to peak singing activity. We averaged corresponding values from measurements at 5‐min intervals for air temperature (TAIR), relative humidity (RELH), and air pressure (PRES) from 2010 to 2019. These average values were labeled as our contemporary weather dataset (Table 1). These variables were used to calculate acoustic atmospheric attenuation (Rossing, 2007).

(1) $$\begin{array}{c} \alpha =f^{2}\left[\left(\frac{1.84\times 10^{-11}}{{\left(\frac{T_{0}}{T}\right)}^{\frac{1}{2}}\times \frac{p_{s}}{p_{0}}}\right)+{\left(\frac{T_{0}}{T}\right)}^{2.5}\times \left(\frac{0.10680e^{-3352/T}\times f_{r,N}}{f^{2}+f_{r,N}^{2}}+\frac{0.01278e^{\frac{-2239.1}{T}}\times f_{r,O}}{f^{2}+f_{r,O}^{2}}\right)\times \frac{\mathrm{Np}}{m\times \mathrm{atm}}\right] \end{array}$$

with *α* as the attenuation coefficient, *f* is the sound frequency, *T* is the absolute temperature of the atmosphere in degrees Kelvin, *T*_0_ is 293.15 K or 20°C, *p*_s_ is the local atmospheric pressure, and *p*_0_ is the reference atmospheric pressure (1 atm = 1.01325 × 10^5^ Pa); *f*_r,N_ is the nitrogen relaxation frequency and *f*_r,O_ is the oxygen relaxation frequency and are calculated by the equations below:

(2) $$f_{r,N}=\frac{p_{s}}{p_{s0}}{\left(\frac{T_{0}}{T}\right)}^{\frac{1}{2}}\times \left(9+280{He}^{-4.17\left[{\left(\frac{T_{0}}{T}\right)}^{\frac{1}{3}}-1\right]}\right)$$

(3) $$f_{r,O}=\frac{p_{s}}{p_{sO}}\left(24.0+4.04\times 10^{4}H\frac{0.02+H}{0.391+H}\right)$$

*H* being the percentage molar concentration of water vapor in the atmosphere or absolute humidity, and is calculated by:

(4) $$H=\frac{\rho_{\mathrm{sat}}r_{h}p_{0}}{p_{s}}$$

with $\rho_{\mathrm{sat}}=10^{C_{\mathrm{sat}}}$ and $C_{\mathrm{sat}}=-6.8346{\left(\frac{T_{0}}{T}\right)}^{1.261}+4.6151$.

**TABLE 1:** Model testing parameters

Weather conditions	Data	Territory size diameter (km)	Song frequencies (kHz)
Contemporary	Average of 2010–2019 ERIC Mesonet data	1	4, 8, 12
Contemporary drought	2011 ERIC Mesonet data	1	4, 8, 12
Mean climate change	Average of 2010–2019 ERIC Mesonet data (+7.5°C, −6%)	1	4, 8, 12
Climate change drought	2011 ERIC Mesonet data (+7.5°C, −6%)	1	4, 8, 12
Medium (climate change drought)	2011 ERIC Mesonet data (+7.5°C, −6%)	1	8
Bad (climate change drought)	2011 ERIC Mesonet data (+7.5°C, −6%)	1.5	8
Worst (climate change drought)	2011 ERIC Mesonet data (+7.5°C, −6%)	3	8

*Note*: We tested multiple combinations of weather conditions, territory sizes, and mean song frequencies with our model. The mean contemporary weather conditions were an average of the 2010–2019 ERIC Mesonet weather data, and the contemporary drought weather data were a subset of the contemporary weather dataset, specifically the year 2011, in which a severe drought occurred in Oklahoma. The mean climate change data were the predicted weather conditions in 2070, and to obtain these values we took the mean contemporary conditions and added 7.5°C to the air temperature (TAIR) and subtracted 6% from the relative humidity (RELH) values. The drought climate change conditions were the predicted extreme weather conditions in 2070 and, to obtain these values, we took the contemporary drought dataset and added 7.5°C to the air temperature (TAIR) and subtracted 6% from the relative humidity (RELH) values. In these conditions, we tested three mean song frequencies (4, 8, and 12 kHz) to determine if frequency would affect neighbor contact rate. For these conditions, we set the territory size to 1 km diameter. For the medium, bad, and worst conditions, we used the climate change drought conditions, kept the mean song frequency to 8 kHz, while varying the territory size diameter to 1 km for the medium conditions, 1.5 km for the bad conditions, and 3 km for the worst conditions.

To simulate contemporary drought conditions, we used a subset of the baseline weather data from the year 2011 when there was a severe drought that affected most of Oklahoma and many neighboring states (Khand et al., 2017; Tadesse et al., 2015). We simulated contemporary drought conditions to determine if vocal activity and the vocal community would change in response to extreme aridity. To simulate the predicted mean climate change conditions in 2070 in which aridity is expected to increase in shrub and grassland habitats, we took the mean weather condition values (TAIR, RELH, and PRES) from 2010 to 2019 ERIC Mesonet station and added 7.5°C to the TAIR and subtracted 6% from the RELH of the mean values (Table 1). These values are based on the predicted climate trends in the North American Southwest for the year 2070 (Huang & Ullrich, 2017). To simulate climate change drought conditions in 2070, we added 7.5°C to the TAIR and subtracted 6% from the RELH from the 2011 ERIC Mesonet weather data (Table 1).

### 2.3 Individual contact percentages

We first ran a simplified version of the model with two individuals and their respective territories to test the effects of multiple song frequencies across multiple territory sizes. We tested 12 frequencies (1–12 kHz) and 60 territory size radii (25–1500 m by 25 m increments) to demonstrate how the different climate conditions listed above would affect the contact rate between two individuals with adjacent territories. We ran this model on the 06/01 date for the mean contemporary, drought, mean climate change, and climate change drought weather data because it was one of the hottest and driest days (based on 2011 TAIR and RELH, respectively) in our weather dataset. We ran this model over five iterations to add variability for the statistical analysis. To determine which frequency and territory radii would be affected by extreme arid weather, we subtracted the contemporary drought and climate change drought results from the contemporary and mean climate change results, respectively (Table 2).

**TABLE 2:** Model completion percentages

Condition	Frequency (kHz)	EWL inclusion/absence	*N*	Percent	SE
Mean contemporary	4	No EWL	5	100.000	0.000
		EWL	5	100.000	0.000
	8	No EWL	5	71.867	0.418
		EWL	5	63.948	0.191
	12	No EWL	5	0.838	0.049
		EWL	5	0.301	0.026
Contemporary drought	4	No EWL	5	99.832	0.020
		EWL	5	99.417	0.060
	8	No EWL	5	40.278	0.367
		EWL	5	30.032	0.095
	12	No EWL	5	3.374	0.093
		EWL	5	2.928	0.152
Mean climate change	4	No EWL	5	100.000	0.000
		EWL	5	100.000	0.000
	8	No EWL	5	91.448	0.262
		EWL	5	79.781	0.360
	12	No EWL	5	12.350	0.349
		EWL	5	1.198	0.028
Climate change drought	4	No EWL	5	99.736	0.042
		EWL	5	97.910	0.138
	8	No EWL	5	50.328	0.222
		EWL	5	29.763	0.294
	12	No EWL	5	5.433	0.166
		EWL	5	0.779	0.091
Medium	8	No EWL	5	49.754	0.291
		EWL	5	30.233	0.268
Bad		No EWL	5	21.034	0.164
		EWL	5	6.913	0.266
Worst		No EWL	5	0.077	0.015
		EWL	5	0.014	0.006

*Note*: These percentages represent the percent of individuals that contacted all six neighbors by the end of the 6‐h model duration.

### 2.4 Population completion percentages

**FIGURE 2:**
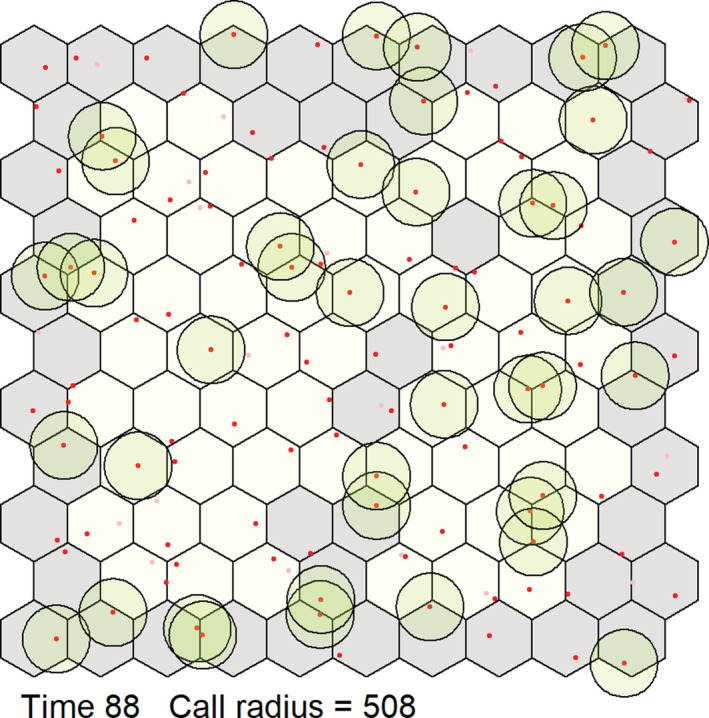
Virtual environment for a population of virtual birds. Each hexagon represents a bird's territory in which the bird moves around. Each bird has six adjacent neighbors, except for the hexagons on the grid edge, which were not included in the final calculations since they could not contact all six neighbors. A bird will either sing, move, or rest until all neighbors were contacted. Birds that have contacted all six neighbors will turn gray and that bird will stop exhibiting behavior for the rest of the day.

This version of the model contained an array of 110 contiguous territories, represented as a hexagonal grid, such that 72 birds (those not on an edge of the array) had six neighbors (Figure 2). We increased the number of birds and territories in a population to determine if the changes in individual contact rates would influence the population‐level communication system. Once a bird contacted all six of its neighbors it would stop singing because it has met the intrasexual condition of defending its territory against its adjacent neighbors. We also tested three different mean song frequencies (4, 8, and 12 kHz), which represented individual bird species to determine if contact rates varied as a function of frequency because these frequencies cover the majority of the avian song frequency bandwidth and 8 kHz is the expected hearing range of most birds (Dooling, 2004). We also ran the model with and without the evaporative water loss (EWL) equation included to determine if water budgets, our measure of individual condition, affected contact rates. In the first time step, the probability of a bird singing, moving, or resting was equal (i.e., 0.33). However, to test if individual conditions affected the probability of these behaviors, these probabilities were subject to change as the model progressed based on how much water had been lost due to thermoregulation. To account for this physiological change, we derived the EWL equation from Albright et al. (2017) for the house finch (*Haemorhous mexicanus*), our model system for singing parameters. Once any bird lost 15% of its body mass due to total EWL (TEWL), it would no longer sing or move and would only rest until the next day because 15% TEWL is considered lethal to the bird (Albright et al., 2017). At the beginning of the next day, the TEWL resets back to zero under the assumption that the birds would recover their water reserves. Both the singing and moving behavior probabilities decreased by half of the TEWL divided by half of 15% of the body mass for each time step.

Each model run consisted of 61 virtual days each of which contained 6 virtual hours or 360 time steps. The 6‐h period represented the dawn chorus and morning singing period, and we disregarded the rest of the day because the morning singing period is usually the highest singing period of the day (Gasc et al., 2017). We evaluated the effectiveness of vocal communication based on the percentage of birds that had successfully contacted all six neighbors at the end of each day. We then averaged these values across all days to generate an overall contact percentage for each of the 360 time steps (i.e., the completion percentage). Birds/territories that were on the edge of the hexagon array were not used to calculate contact rates as they had fewer than six neighbors. Hence, completion percentages were calculated based on the 72 inner territories (Video S1).

### 2.5 Statistical analysis

To analyze how different environmental conditions, frequencies, and territory sizes would affect individual contact rates, we developed a linear model (LM) using the lme4 package in the R statistical software (Bates et al., 2014). This model included the weather conditions, mean song frequency, and the inclusion or absence of the EWL equation on bird contact percentage. We conducted regression diagnostic tests and the residuals were somewhat normally distributed for this model. We also conducted an LM on the population‐level, completion percentage after conducting regression diagnostic tests and finding somewhat normally distributed residuals for the population contact percentages. We averaged the total number of birds that contacted a neighbor for each time step across the 61 days within each iteration, which gave us the mean completion percentage across our model duration. We analyzed the interactions among weather conditions, frequency, and the inclusion or absence of the EWL equation on the territory completion percentages. For both the individual‐ and population‐level contact analysis, we conducted a three‐way ANOVA and a Tukey post hoc test on the three‐way interactions using the “car” and “multcomp” packages, respectively (Fox & Weisberg, 2018; Hothorn et al., 2008), to determine which combinations of variables were significantly different from each other. All statistical analyses were done in R v4.0.4 (R Core Team, 2021).

## 3 RESULTS

### 3.1 Individual effects

**FIGURE 3:**
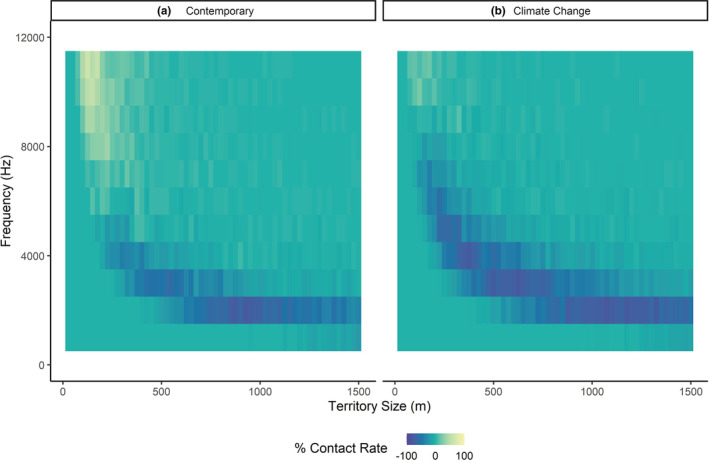
Heatmaps of frequencies affected by extreme temperatures across different territory sizes. We applied the ABM to multiple territory sizes ranging from 25 m radius territories to 1500 m radius territories across the audible bird song frequencies for the differences in drought versus contemporary weather data (a), and the differences in climate change drought versus climate change weather data (b). Cooler colors represent frequencies and territory sizes that would lead to fewer birds successfully contacting all neighbors under extreme conditions in both the extreme and climate change drought data, suggesting that selection may drive bird populations toward smaller territory sizes and higher frequency songs.

According to the LM results, increasing frequency and territory size led to decreases in the mean contact percentages between the contemporary drought conditions and the mean contemporary conditions (*B* = −8.81e07 ± 24.05e‐07 SE, *t* = 2.175, *p* = .030) and between the mean climate change conditions and mean contemporary conditions, although this trend was not significant (*B* = −7.22e‐07 ± 4.05e‐07 SE, *t* = −1.782, *p* = .075), indicating fewer contacts between neighbors as song frequency and territory size increased. These effect sizes were very small, which suggests that territory size and frequency may not be the most important factors in determining vocal contact between neighbors. The nature of these simulations would also produce significant results because of the way the simulation was designed. The ANOVA demonstrated that there were significant differences between the weather conditions after accounting for variations in frequency and territory size (*F* = 4.084, *df* = 3, *p* = .007). The Tukey post hoc test on weather conditions demonstrated that compared to the mean contemporary conditions, contact percentages significantly decreased in the mean contemporary drought conditions by ~17% (*B* = −17.356 ± 2.615 SE, *t* = −6.638, *p* = .001) and in the mean climate change drought conditions by ~16% (*B* = −16.469 ± 2.615 SE, *t* = −6.299, *p* < .001). The mean climate change conditions had higher contact percentages than the mean contemporary conditions by ~6% but this trend was not significant (*B* = 6.059 ± 2.615 SE, *t* = 2.317, *p* = .094). To determine which frequencies would be affected by the extreme arid conditions, we subtracted the results of the simple model under the mean contemporary conditions from the drought conditions results (Figure 3a) and the mean climate change conditions from the climate change drought conditions results (Figure 3b) to demonstrate how extreme conditions affect mean song frequencies under different territory sizes. Smaller territory sizes with higher frequencies had higher contact percentages compared to larger territories with lower frequencies.

### 3.2 Population effects—weather conditions

**FIGURE 4:**
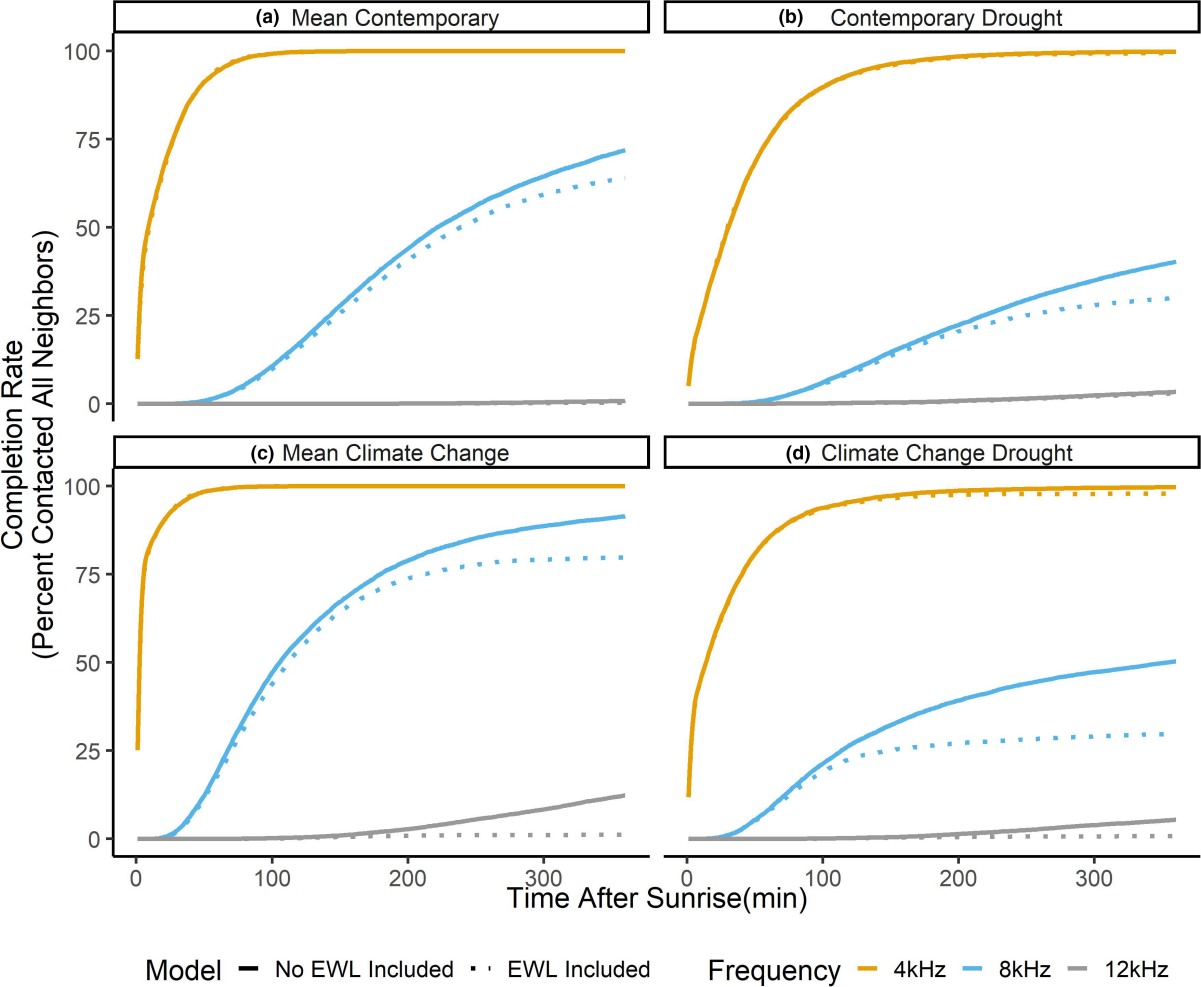
Population completion percentages across four different environment conditions: (a) mean contemporary, an average of air temperature (TAIR), relative humidity (RELH), and air pressure (PRES) from the ERIC Mesonet station from 2010 to 2019. (b) Contemporary drought, the weather data from the 2011 drought from the ERIC Mesonet station. (c) Mean climate change, 7.5°C TAIR increase, and 6% RELH decrease to the mean contemporary data. (d) Climate change drought, 7.5°C TAIR increase, and 6% RELH decrease to the contemporary drought weather data. Three frequencies that span the songbird frequency bandwidth were tested (4 kHz: Orange, 8 kHz: Blue, 12 kHz: Gray). Models without (solid) and with (dotted) the evaporative water loss (EWL) equation are included.

The ANOVA demonstrated that there were significant effects of weather conditions, frequency, and inclusion/absence of the EWL equation on completion percentages (*F* = 13,146, *df* = 23, *p* < .001). Based on the Tukey post hoc tests, mean completion percentages between the contemporary drought conditions and mean contemporary conditions (Figure 4a) decreased for 4 and 8 kHz by ~7% and ~16%, respectively (Figure 4b, 4 kHz: *B* = −7.209 ± 0.494 SE, *t* = −14.584, *p* = <.001; 8 kHz: *B* = −16.438 ± 0.494, *t* = −33.253 SE, *p* < .001). The climate change drought conditions also had lower completion percentages for 4 and 8 kHz by ~3% and ~4%, respectively (Figure 4d, 4 kHz: *B* = −3.423 ± 0.494 SE, *t* = −6.924, *p* < .001; 8 kHz: *B* = −4.093 ± 0.494 SE, *t* = −8.28, *p* < .001). The completion rates for the mean climate change conditions were higher than the mean contemporary conditions for all frequencies by ~13%, ~26%, and ~3%, respectively (Figure 4c, 4 kHz: *B* = 3.091 ± 0.494 SE, *t* = 6.252, *p* < .001; 8 kHz: *B* = 26.361 ± 0.494 SE, *t* = 53.327, *p* < .001; 12 kHz: *B* = 3.445 ± 0.494 SE, *t* = 6.970, *p* < .001). Completion rates for 12 kHz were also significantly lower between the climate change drought and mean climate change conditions by ~2% (12 kHz: *B* = −1.948 ± 0.494 SE, *t* = −3.942, *p* < .018).

### 3.3 Population effects—frequency

Completion percentages decreased with increasing frequency for mean contemporary by ~60% when increasing from 4 to 8 kHz, and ~95%, when increasing from 4 to 12 kHz (8 kHz: *B* = −59.901 ± 0.494 SE, *t* = −121.178, *p* < .001; 12 kHz: *B* = −95.014 ± 0.494 SE, *t* = −192.209, *p* < .001). Contemporary drought completion percentages decreased with increasing frequency by ~69% when increasing from 4 to 8 kHz, and ~7% when increasing from 4 to 12 kHz (8 kHz: *B* = −69.30 ± 0.494 SE, *t* = −139.846, *p* < .001; 12 kHz: *B* = −86.945 ± 0.494 SE, *t* = −175.886, *p* < .001). Mean climate change completion percentages decreased with increasing frequency by ~37% when increasing from 4 to 8 kHz and ~95%, when increasing from 4 to 12 kHz (8 kHz: *B* = −36.631 ± 0.494 SE, *t* = −74.103, *p* < .001; 12 kHz: *B* = −94.659 ± 0.494 SE, *t* = −191.491, *p* < .001). Completion percentages decreased with increasing frequency in the climate change drought conditions by ~61% when increasing from 4 to 8 kHz and ~90%, when increasing from 4 to 12 kHz (8 kHz: *B* = −60.572 ± 0.494 SE, *t* = −122.534, *p* < .001; 12 kHz: *B* = −90.094 ± 0.494 SE, *t* = −182.256, *p* < .001).

### 3.4 Population effects—evaporative water loss

When we included the EWL equation in the model, EWL significantly reduced completion rates at 8 kHz for contemporary conditions by ~3% (8 kHz: *B* = −2.829 ± 0.494 SE, *t* = −5.724, *p* < .001), the contemporary drought conditions by ~3% (8 kHz: *B* = −2.959 ± 0.494 SE, *t* = −5.987, *p* < .001), mean climate change conditions by ~5% (8 kHz: *B* = −5.138 ± 0.494 SE, *t* = −10.395, *p* < .001), and climate change drought conditions by 9% (8 kHz: *B* = −9.733 ± 0.494 SE, *t* = −19.689, *p* < .001). EWL also significantly reduced contact rates for 12 kHz for the mean climate change conditions by ~3% (12 kHz: *B* = −2.970 ± 0.494 SE, *t* = −6.007, *p* < .001).

### 3.5 Population effects—territory size

**FIGURE 5:**
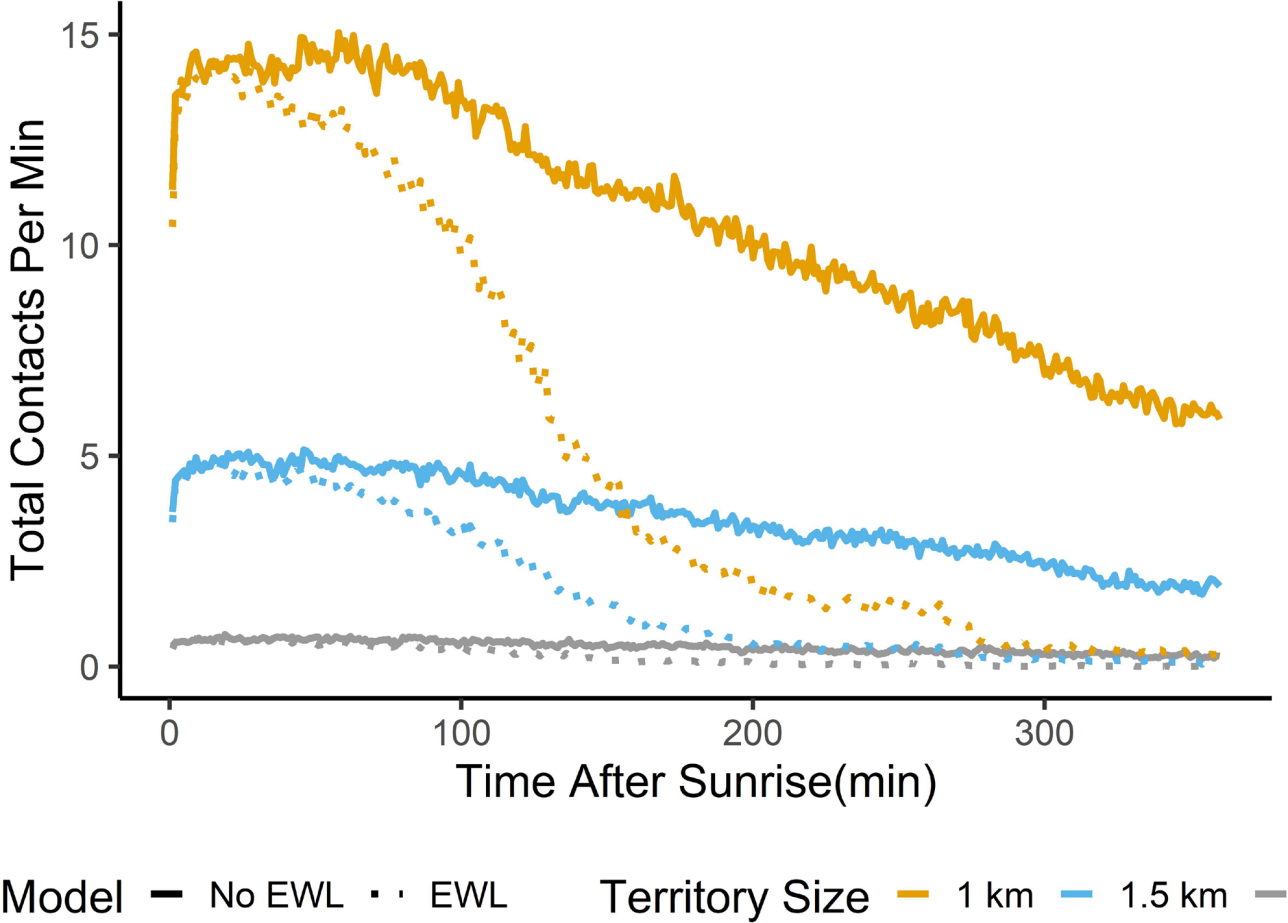
Total contacts decrease as territory size increases under extreme climate change conditions (i.e., the “worst case scenarios”) for 8 kHz. A total number of contacts is represented on the *y*‐axis and time (min) is represented on the *x*‐axis. We tested the model under these conditions without (solid) and with (dotted) the evaporative water loss (EWL) equation.

Since the 8 kHz frequency demonstrated the most significant effects, we tested this frequency at various territory size diameters (1.0, 1.5, and 3.0 km) under the climate change drought (Figure 5). The ANOVA demonstrated that the interaction between territory size and inclusion/absence of the EWL equation had significant effects on completion percentages (*F* = 4019.6, *df* = 5, *p* < .001). Completion percentages decreased significantly as territory size increased from 1.0 to 1.5 km by ~21% (*B* = −21.328 ± 0.283 SE, *t* = −75.311, *p* < .001), increased from 1.0 to 3.0 km by ~31% (*B* = −30.944 ± 0.283 SE, *t* = −109.266, *p* < .001), and increased from 1.5 to 3.0 km by ~10% (*B* = −9.629 ± 0.283 SE, *t* = −34.000, *p* < .001). When EWL was included, completion percentages decreased significantly as territory size increased from 1.0 to 1.5 km by ~18% (*B* = −17.608 ± 0.283 SE, *t* = −62.174, *p* < .001), increased from 1.0 to 3.0 km by ~22% (*B* = −21.953 ± 0.283 SE, *t* = −77.517, *p* < .001), and increased from 1.5 to 3.0 km by 4% (*B* = −4.346 ± 0.283 SE, *t* = −15.345 *p* < .001).

## 4 DISCUSSION

Based on the ABM results, we concluded extreme arid conditions would lead to reduced contact rates and an altered avian soundscape for birds with mid‐to‐high mean song frequencies. While we found support for the FAB1 hypothesis, wherein reduced physiological condition (i.e., high TEWL) led to lower completion rates for the 8 kHz mean song frequency under contemporary drought and climate change drought conditions, the majority of our results supported the FAB2 hypothesis, indicating that species' specific soundscapes are dependent on fixed, species' specific traits (i.e., mean song frequency and territory size). This trait dependence means that with increasing aridity, certain species will be excluded due to reduced efficacy of the vocal communication system. This species loss will change the community composition of singing songbirds, which can be an indicator of community health (O'Connell et al., 2000). Our model demonstrates that under high aridity levels, the vocal communication system is not as efficient in transmitting and receiving information as under less arid conditions. Therefore, the population cannot maintain acoustic contact with each other, which could lead to changes in territorial boundaries and potential mating opportunities. These individual consequences could lead to population‐level effects such as a decline in population size, which could alter the community soundscape. Alternatively, species can adjust their singing behavior to accommodate for these extreme conditions by producing signals with optimal transmission distance or by having a more gregarious social system, however, not all species would be able to display behavioral flexibility or developmental plasticity accordingly.

We found from the population‐level analysis that species‐specific traits such as mean song frequency were the main determinant in maintaining contact with their adjacent neighbors; specifically, species with low mean song frequencies were still able to contact all neighbors across all the different weather conditions tested (Figure 4). Low‐frequency acoustic signals are still transmitted in desert environments later in the day, while high‐frequency acoustic signals are attenuated at a much higher rate under the same conditions (Henwood & Fabrick, 1979). Species with fixed vocalization frequencies, such as suboscines, which have innate vocalizations, will be more likely affected by the increasing arid conditions because they cannot adjust their vocalization frequency for higher transmission distance (Ríos‐Chelén et al., 2012). Suboscine species with high‐frequency vocalizations would have difficulty maintaining vocal contact with neighbors, as seen in a study on vermillion flycatchers (*Pyrocephalus obscurus*), which did not shift their song frequencies in response to high levels of anthropogenic noise (Ríos‐Chelén et al., 2018). Bird species with more plastic singing behavior may be able to adjust their song frequency to increase transmission in nature. White‐throated sparrows (*Zonotrichia albicolis*) adjust song characteristics such as song duration, higher minimum frequencies, and narrower‐frequency bandwidths under high periods of noise (Lenske & La, 2014). Male black‐capped chickadees (*Poecile atricapillus*) can shift their song frequencies up when masking noise is played (Goodwin & Podos, 2013). While we found limited evidence for the FAB1 hypothesis as seen in reduced contact percentages with the inclusion of the EWL equation for mid‐to‐high mean song frequencies (Figure 4), other studies demonstrated that avian physiology can reduce singing behavior, especially under high temperatures (Coomes & Derryberry, 2021; McGrann & Furnas, 2016). Another study demonstrated that willow warblers (*Phylloscopus trochilus*) increased metabolic rate and thermoregulation costs while singing (Ward & Slater, 2005), so one could assume that increased thermoregulatory needs and high TEWL could lead to decreased vocalization behavior.

Species whose singing and movement behaviors are fixed are more vulnerable to soundscape degradation associated with increased aridity. As song transmission distance decreases, maintaining contact rates will require some combination of lowering mean song frequency, increasing song volume, and altering the timing and intensity of singing to correspond with favorable conditions. For species that are flexible in their singing behavior, they will need to create new song types to sing in an arid environment (which supports the acoustic adaptation hypothesis), or change their peak singing time to periods of low aridity (which supports the acoustic niche hypothesis) (Krause, 1993; Morton, 1975). Selection for more transmissible songs in degraded environments has been noted in multiple species. North American warblers (Parulidae) decrease their signal frequency bandwidth (max frequency span within a note) and increase the signal length under high atmospheric attenuation (Snell‐Rood, 2012). Southern house wrens (*Troglodytes musculus*) had lower song amplitude at high atmospheric attenuation conditions (Sementili‐Cardoso & Donatelli, 2021). In many bat species, which vocalize in ultrasonic frequencies, warmer climates have led to higher‐frequency calls that attenuate due to high temperatures and low humidity (Luo et al., 2014), which could lead to the selection of lower‐frequency vocalizations and longer vocalizations to transmit information to the intended receivers. This selection for certain song syllables and song types could lead to the selection of low‐frequency, long songs, which would lead to the decrease in song‐type diversity in the avian soundscape. Species with higher‐frequency songs may relocate to habitats that have more favorable acoustic properties or reduce singing under hotter periods of the day (Diepstraten & Willie, 2021; McGrann & Furnas, 2016). Alternatively, selection can favor increased behavioral plasticity for birds to continue to sing in arid environments. Species that are not able to adjust their singing behavior can no longer maintain a vocal communication system in arid environments and will need to move to a more suitable habitat.

While our model did not account for behavioral plasticity or intraspecific variation for individual species, this exclusion does not necessarily negate support for the FAB2 hypothesis. Completion rates did shift under the different climate scenarios, but the frequency was the deciding factor in the difference in completion rates (Figure 4). While plastic behavior is shown in many species, plasticity itself can be fixed, unless selection acts on it to increase (Crispo, 2007). It has been documented that certain species' singing behavior is flexible in disturbed habitats, particularly in habitats with high anthropogenic noise. Many urban birds such as oregon juncos (*Junco hyemalis oreganus*; Reichard et al., 2019), great tits (Slabbekoorn & den Boer‐Visser, 2006), and white‐crowned sparrows (Derryberry et al., 2016) shifted their minimum song frequency to transmit signals above the low‐frequency anthropogenic noise. Other species, like the vermillion flycatchers sing longer songs during noisy habitats (Ríos‐Chelén et al., 2013) and serins (*Serinus serinus*) increase vocal activity during noisy periods (Díaz et al., 2011). Birds in arid environments may also adjust their singing behavior by singing during the morning periods when the sound transmission is the highest. Altitudinal migrants and resident bird species reduced singing activity when temperatures were high, but neotropical migrants retained their singing activity even though the risk of heat stress was high (McGrann & Furnas, 2016). With behavioral plasticity included, the FAB2 still retains support because the limits of plasticity can be limited by species‐specific interactions.

If territorial songs are unable to propagate and reach their intended receivers, then the efficiency of the song decreases and the cost of singing increases (Wiley, 1998). This reduced efficacy could result in individuals not being able to find mates due to incomplete information reaching the receiver, or potential mates preferring non‐degraded songs. In many songbird species, females prefer certain song types over others. In habitats with high levels of anthropogenic noise, an example of a disturbing soundscape, ovenbirds (*Seiurus aurocapilla*) suffered lower rates of pairing than in quieter habitats (Habib et al., 2006). Wild male zebra finches (*Taeniopygia castanotis*) that sing longer and higher pitched songs predicted hatching success and the number of genetic offspring surviving (Woodgate et al., 2012). Preference can also play a factor in reduced population recruitment; female Lincoln's sparrows (*Melospiza lincolnii*) have a higher preference for male songs sung in colder temperatures versus warmer temperatures (Beaulieu & Sockman, 2012). On the opposite spectrum, male pied flycatchers (*Ficedula hypoleuca*) singing in cold temperatures are preferred less by female pied flycatchers than males singing in warmer temperatures (Slagsvold & Dale, 1994). Warmer temperatures could also lead to potential mates preferring conspecific songs over heterospecific songs (Coomes et al., 2019), which could lead to missed mating opportunities and ultimately reduced population recruitment.

In addition to a reduced preference for certain song types, as aridity increases, resources like food and water will decrease in abundance, and birds will need to expand their territories to have the necessary resources to survive and reproduce (Dean et al., 2009; Khoury & Boulad, 2010). This increase in territory size will lead to increased energetic demand for patrolling territories, especially if the vocal signals used to maintain territory boundaries no longer reach their intended receivers. If vocal activity decreases, then individuals will need to increase territorial movement behavior to actively defend their territories from intruders. Increasing resource needs when resources are already low would push individuals past their breaking point (McKechnie et al., 2012), and while behavioral flexibility (short‐term behavioral plasticity) would provide quick relief, the increased allostatic load would be too much for some species to adapt and develop non‐reversible plasticity (i.e., developmental plasticity) to deal with increasingly extreme conditions (Wingfield et al., 2017). Outside of mating and reproduction, for social species, the reduced soundscape under arid conditions could lead to reduced flock foraging behavior (Safriel, 1990) as calls may not reach conspecifics. The reduced transmission of predator alarm class could have community‐level effects, such as elevated depredation events, due to multiple species listening to heterospecific alarm calls (Grade & Sieving, 2016). Pair‐bonded individuals will not be able to coordinate parental provisioning or produce effective alarm calls to warn of predators (Rose et al., 2020) if the vocal signals are degraded. Territorial behaviors may decrease if degraded signals reach neighbors because birds rely on ranging or auditory cues to evaluate the distance of a conspecific. If a trespassing neighbor's song is degraded by high atmospheric attenuation, then the focal individual may not respond aggressively enough to the trespassing neighbor because the focal individual thinks the trespasser is farther away than it really is (Farina, 2014; Fotheringham et al., 1997). Alternatively, aggressive territorial behaviors may increase due to competition over decreasing resources (Samplonius & Both, 2019). Many bird species use vocalizations to defend territories against rival mates and prevent extra‐pair paternity (Mace, 1987). Males that move more could suffer from extra‐pair fertilizations occurring on their territory. If the male is unable to defend the territory, territoriality behavior could become ineffective. Alternatively, individuals that are unable to defend a territory or no longer have access to a territory could become helpers on an existing territory of a more dominant/successful individual. With the degraded soundscape leading to fewer mating opportunities and increasing aridity leading to limited resources unable to support multiple breeding individuals, cooperative helpers may assist dominant individuals to patrol territories and help with nestling provisioning (Koenig & Dickinson, 2004). A direct effect of reduced resources leading to larger territories is smaller population densities, which coupled with attenuated song types/syllables could lead to the loss of vocal culture or reduced vocal repertoires. A decrease in population size and density resulted in a decrease in vocal culture in regent honeyeaters (*Anthochaera phrygia*). Male regent honeyeater songs in 2011 were shorter and contained fewer syllables than songs in 1968 due to habitat fragmentation (Valderrama et al., 2013). This decrease in vocal culture led to reduced female pairing (Crates et al., 2021). Song‐type diversity may also decrease due to cultural selection in tandem with natural selection if certain signals are not learned by the next generation (Searcy & Nowicki, 2005). Oscines or birds that learn song types from a tutor would only be exposed to low‐frequency, long syllable song types in an arid, degraded soundscape and once they mature their offspring will learn those song types as well. One study demonstrated that young Carolina wrens (*Thryothorus ludovicianus*) prefer to learn undegraded songs than degraded songs (Morton et al., 1986), and under the predicted extreme aridity conditions, there may be fewer degraded song types to choose from. To reduce degradation, birds may position themselves higher in tree canopies (Mathevon et al., 2005), which may expose birds to new ecological niches. Our results demonstrated that higher mean song frequencies would be less likely to transmit to adjacent neighbors, indicating that high‐frequency signals could be lost in arid songbird communities because they will not be heard by young birds. Alternatively, the singing activity could potentially increase due to geophonies (i.e., sounds from the natural environment) decreasing due to dry riverbeds (Krause & Farina, 2016). Regardless, species living in variable conditions and unpredictable environments will need to learn and invent new syllables and song types in order to communicate to their intended receivers (Botero et al., 2009; Laiolo, 2008; Laiolo & Tella, 2006), which could lead to an increase in syllable and song‐type diversity.

Changing song characteristics and song diversity would be an example of adaptation or a plastic response. Increasing phenotypic plasticity can help species continue to function in extreme environments, and these extreme environments can therefore select more plastic traits that reduce trait costs (Chevin & Lande, 2010; Hoffmann & Parsons, 1993). If the extreme environments continue to persist, organisms may develop non‐reversible plasticity, which could lead to trait adaptation (Wingfield et al., 2017). While the selection may favor plastic traits, plasticity is dependent on other traits (e.g., behavioral syndromes) that may limit behavioral expression, which could prevent species from expressing the optimal trait in a given context (Lande, 2009). Plastic responses can be adaptive if those plastic responses in mild conditions are genetically correlated with responses in extreme conditions (Chevin & Hoffmann, 2017). Species with these correlated, plastic responses may have built‐in climate resilience, which would lead to better chances of survival under increasingly extreme environments (Chevin et al., 2010).

Increasing aridity may completely alter soundscapes which can have individual‐, population‐, and community‐level impacts. The acoustic niche hypothesis states that species will occupy individual niches to avoid frequency or temporal overlap (Krause, 1993). With increased aridity changing the optimal times to sing, increased temporal overlap may occur between species that before did not compete for the same frequency range (Krause, 2012). The highest acoustic activity occurs during the dawn choruses, and while multiple factors like physiology (Thomas et al., 2002; Thomas & Cuthill, 2002), light intensity (Berg et al., 2006), and social factors (Krebs & Kacelnik, 1983) affect the dawn chorus activity, if aridity negatively impacts the sound propagation characteristics during the day, then multiple species will compete for the same temporal space during the dawn chorus (Krause, 1987). With selection favoring lower‐frequency songs and with a smaller optimal window to produce high‐frequency songs in arid environments, species that are unable to shift their song frequencies or cannot produce new songs will need to relocate to more suitable habitats. This relocation could lead to interspecific conflict between native species and the relocating species that use the same acoustic niche (Farina et al., 2013), and potential divergence of species as the colonizing species begins to adapt to the new acoustic environment (Cardoso & Price, 2010). This change in community composition could lead to an avian soundscape dominated by functional diversity (i.e., low‐frequency songs in large territories or medium‐frequency songs in small territories) rather than phylogenetic diversity (Gasc et al., 2013).

Soundscapes can represent the health of an environment if acoustical niches correlate with ecological niches of vocal animals (Farina et al., 2011; Fuller et al., 2015; Gage & Axel, 2014; Kasten et al., 2012). Soundscapes can be used to detect early signs of bird stress or disturbance related to habitat or climate changes (Doser et al., 2020; Sueur & Farina, 2015). Since the 1990s, the avian community soundscape has become more homogeneous, acoustic diversity has decreased, and soundscape intensity has declined in northern and eastern North America (Morrison et al., 2021). Degrading soundscapes could lead to reduced perceived ecosystem value for many habitats (Ferraro et al., 2020). Humans have increased perceived ecosystem value if a habitat sounds more “natural” (Francis et al., 2017). Ecosystem services can be enhanced by making a habitat sound more natural, which in turn could lead to an increase in conservation support (Levenhagen et al., 2021). Protecting a soundscape is vital for adding ecosystem value to a habitat so we can advocate for ecosystems for the public's benefit.

Our model demonstrated how changes in individual singing and movement behaviors due to extreme aridity can lead to an altered avian soundscape at the population level. This change in the soundscape could lead to certain species adapting and continuing to sing during these increasingly arid conditions, while other species may reduce their vocalizing behavior. Identifying which species would suffer from this altered communication system can potentially be helpful in creating mitigation strategies such as adding supplemental water resources or creating artificial shade refugia to help reduce the impact of increasing aridity on avian populations. Further analyses across multiple species are needed to determine how increasing aridity will affect an avian soundscape at the community level.

## AUTHOR CONTRIBUTIONS

**Meelyn M. Pandit:** Conceptualization (supporting); data curation (equal); formal analysis (equal); investigation (equal); methodology (equal); project administration (equal); software (supporting); validation (lead); visualization (lead); writing – original draft (lead); writing – review and editing (equal). **Eli S. Bridge:** Conceptualization (supporting); data curation (equal); formal analysis (equal); methodology (lead); software (lead); writing – review and editing (equal). **Jeremy D. Ross:** Conceptualization (lead); formal analysis (equal); methodology (equal); project administration (lead); supervision (lead); writing – review and editing (equal).

## CONFLICT OF INTEREST

We declare no conflicts of interest.

### OPEN RESEARCH BADGES

This article has earned Open Data and Open Materials badges. Data and materials are available at https://doi.org/10.5061/dryad.r7sqv9sg8.

## Supporting information


Video S1

## Data Availability

We provide all R scripts of the agent‐based model, the weather data, and the statistical analysis scripts can be found on Dryad: https://doi.org/10.5061/dryad.r7sqv9sg8.
